# Traditional birth attendants’ knowledge, preventive and management practices for postpartum haemorrhage in Osun State, Southwestern Nigeria

**DOI:** 10.1038/s41598-023-39296-y

**Published:** 2023-07-29

**Authors:** Deborah Tolulope Esan, Olabisi Helen Ayenioye, Paul Oladapo Ajayi, Adewale Allen Sokan-Adeaga

**Affiliations:** 1 Department of Nursing Science, College of Health Sciences, Bowen University, P.M.B. 284, Iwo, Nigeria; 2 Department of Nursing Science, College of Medicine and Health Sciences, Afe Babalola University, Ado-Ekiti, Nigeria; 3 Department of Community Medicine, Faculty of Clinical Sciences, Ekiti State University, Ado-Ekiti, Ekiti State Nigeria; 4 Department of Environmental Health Science, Faculty of Basic Medical Science, Ajayi Crowther University, Oyo, Oyo State Nigeria

**Keywords:** Health care, Medical research

## Abstract

Traditional birth attendants (TBAs) have become an integral part of the workforce providing delivery services in Nigeria due to the limited number of skilled birth attendants and cultural preferences. This study assessed the knowledge, management and preventive practices regarding postpartum haemorrhage (PPH) among TBAs in selected communities in Osun State, Southwest Nigeria. The study employed a descriptive cross-sectional study design and recruited 260 TBAs in four communities in Osun State. Data were collected by means of an adapted semi-structured questionnaire. Data were analysed using SPSS version 23 and summarized using descriptive and inferential statistics (chi-square and logistic regression) with the level of significance set at *p *< 0.05. The findings indicated that most (71.4%) of the TBAs were cleric, while others were herbalist (28.6%). Although the majority (76.4%) of the TBAs had good knowledge of the causes and warning signs of PPH, a high percentage (69.3%) of TBAs had poor management practices, while 114 (64.1%) TBAs had inadequate preventive practices. Notably, almost none of the participants practised active management of the third stage of labour; the majority of TBAs did not administer any uterotonic drugs to the mother, nor did they deliver the placenta by controlled cord traction. Gender (*P* = 0.029), educational level (*P* = 0.035) and average number of births per month (*P* = 0.001) significantly influenced TBAs’ *management* practices. Similarly, the TBA type (*P* < 0.001), average number of births per month (*P* = 0.003) and experience with formal training (*P* = 0.005) showed significant associations with TBAs’ *preventive* practices. Furthermore, TBAs’ preventive practices towards PPH were influenced by the TBA type (OR: 4.23; 95% CI 1.64–10.90). TBA management practices were also influenced by the TBA type (OR: 4.42; 95% CI 2.03–9.61). Traditional birth attendants in this study had poor management and poor preventive practices for postpartum haemorrhage.

## Introduction

Globally, maternal mortality is decreasing, yet an estimated 300,000 maternal deaths occur annually in developing countries^1^. The single major cause of maternal death is postpartum haemorrhage (PPH)^1^. PPH is defined by the World Health Organization as blood loss of 500 ml or more within 24 h after birth^2^. PPH is a significant cause of maternal mortality in developing countries and the main cause of maternal deaths worldwide (approximately^2^ one-quarter)^2^. Nearly all deaths resulting from PPH occur within the first 24 h after birth. The majority of these could be avoided through the use of prophylactic uterotonics during the third stage of labour and by timely and appropriate management^2^.

In developing countries where the majority of maternal death occurs, multiple causes include haemorrhage, obstructed labour, sepsis, eclampsia/preeclampsia, anaemia, inadequate human resources for health, delay in seeking care, inadequate equipment, lack of ambulance transportation, and delay in referral services^3,4^. Approximately 94% of all maternal deaths occur in low- and lower-middle-income countries^4^, including Nigeria. Approximately half of these maternal deaths are referred from traditional birth attendants (TBAs) and mission houses^3^. Due to inadequate human resources coupled with a massive brain drain of health care workers in the health sector in low- and lower-middle-income countries^3,5^, there is a massive deficit of human resources for health^5^. Due to this deficit in most developing countries, TBAs remain a major part of maternal and child health care in resource-poor settings such as Nigeria^6^.

A consensus emerged in the late 1990s among leaders in global maternal health that TBAs should no longer be trained in delivery skills and should instead be trained as promoters of facility-based care^7^. However, traditional birth attendants have remained in most developing countries due to their acceptability (cultural, norms, financial and spiritual), accessibility, and availability as well as the poverty level, poor female education and weak female empowerment^7,8^. A TBA, according to the World Health Organization, is “a person who assists a mother during childbirth and who initially acquired her skills by delivering babies herself or through apprenticeship to other traditional birth attendants”^95^.

The topic of this study is largely under researched and poorly understood. However, in developing countries, TBAs continue to play a key role in maternal and child health. If Sustainable Development Goals 3 and 3.1 are to be achieved by 2030 in these parts of the world, the input of TBAs must not be neglected, and the approach should be inclusive and supportive. This study aims to identify the knowledge gap among TBAs for PPH and the preventive practices and management practices for PPH among TBAs. The study also investigates the predictors of good preventive practices and management practices among TBAs.

## Methods

### Research design

This study employed a descriptive cross-sectional design designed to assess the management and preventive practices of traditional birth attendants regarding postpartum haemorrhage.

### Description of study area

**Figure 1:**
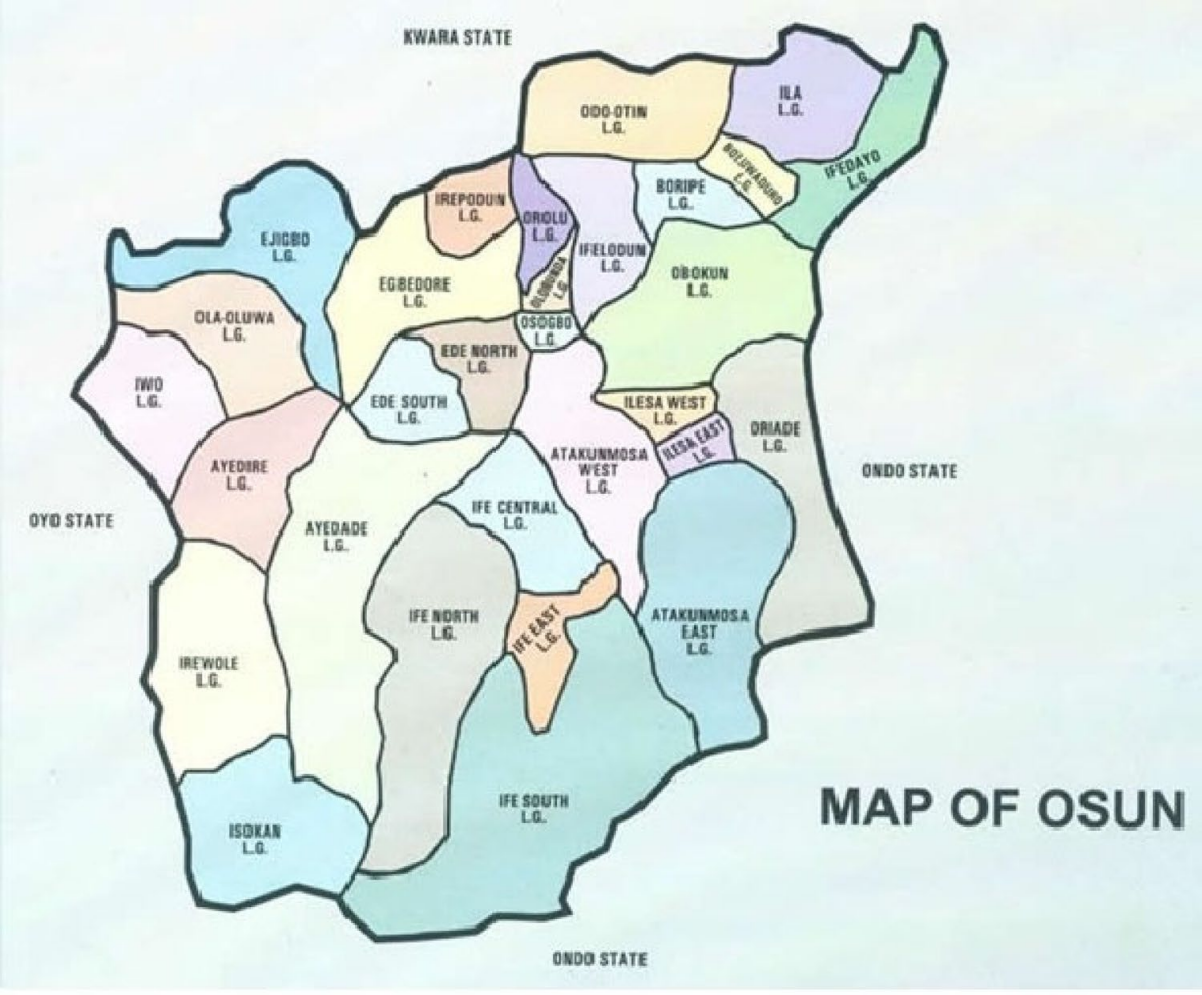
Map of Osun State in Southwestern Nigeria.

The study was carried out in Osun state, south-western Nigeria and it’s organized politically into 30 local government areas and three senatorial districts, as illustrated in Fig. 1^10^. Osun has 3,423,535 residents, 1,740,619 of whom are male and 1,682,916 of whom are female, according to the 2006 National Population Census (National Population Commission. 2006). Osun State's population grew by 3.11% per year between 2006 and 2011, according to estimates. According to this annual growth rate, Osun’s population is currently estimated to be approximately 5,521,901 in 2023^11^.

The state health sector came into being during the creation of the state on 27th August 1991 with two (2) agencies, the Ministry of Health and the Hospitals Management Board, as the administrative and supervisory bodies controlling the activities of the sector. Furthermore, the number of health care facilities has increased as follows: 876 primary health care (PHC) facilities, 57 secondary health care facilities, and 2 tertiary health institutions, Obafemi Awolowo University Teaching Hospital Complex (OAUTHC) and Osun State University Teaching Hospital^12^. Maternal mortality remains persistently high in Osun State with no significant improvement; accounting for 32% of all deaths among women of reproductive age^10,13,14^. This menace is largely due to suboptimal uptake and quality of antenatal care, low utilization of TBAs and skilled birth attendants (SBAs) (38%), high rates of home deliveries, poor quality of delivery services, limited access to emergency obstetric care services and adverse reproductive behaviours. TBAs/SBAs have 92% coverage across Osun State^12,15,16^.

### Baseline information

TBAs are persons (usually women) who assist mothers at childbirth and who initially acquire their skills by delivering babies themselves (self-taught) or by working with other TBAs (apprenticeship)^17^. They are traditional, independent (of the health system), non-formally trained, community-based providers of care during pregnancy, childbirth and the postnatal period^18^. When TBAs receive any form of formal midwifery training, they are referred to as trained TBAs (TTBAs). There are two types of TBAs in Osun State and the country (Nigeria): *faith-based* and *private practitioners*. Faith-based TBAs operate **1.** in “faith homes‟, most of which are affiliated with churches and a few with mosques and are often referred to as *cleric TBAs* and **2.** others with traditional healing institutions, who are referred to as *herbalist TBAs*^19^. TBAs’ protocols in Nigeria are prepared and imposed by the National Primary Health Care Development Agency (NPHCDA), an agency under the Ministry of Health. The agency ensures compliance through periodic monitoring by its various arms of the local government primary health care departments (LGPHCDs)^20^.

In Osun State, Nigeria, the Ministry of Health as well as the various LGPHCDs are involved in the training and retraining of TBAs. At the local government level, a training session lasts for 3 weeks, while retraining lasts for a week. A trained TBA is expected to have retraining at least yearly. The training cost is borne by the participants. TBAs are trained in correct knowledge of the function, dosing and timing of administration of misoprostol and oxytocin and the use of the delivery kits. Upon completion of training, each TBA is given a delivery kit, misoprostol, oxytocin, antibiotics, analgesics, Fleet enemas and other drugs. Each local government has a TBA programme officer who coordinates the training programme and supervises their practices. The programme officer in most cases is a chief nursing officer with the local government PHC department. Trained TBAs are registered with the local government and are expected to meet with the programme officer on a monthly basis for a review of their activities. There is no precise figure for the total number of registered TBAs in the state due to poor data management, a common problem that afflicts all parastatals in the country^10,15^.

### Study setting

The study was conducted in four major towns in Osun State, southwest Nigeria: Ilesa, Osogbo, Ile-Ife and Gbongan.

*Gbongan* is a significant town in Nigeria's Osun State. It serves as the administrative centre for the Aiyedaade Local Government Area. Its geographical coordinates are 7° 28′ 0″ North, 4° 21′ 0″ East. Ayedaade falls under the Irewole Federal Constituency and under the Osun West Senatorial District.

*Ile-Ife,* also known as Ife, Ife-Lodun, or the Kingdom of Ife, is a historic Yoruba metropolis in Osun State, Nigeria. The settlement is located at the crossroads of the Ibadan (40 miles [64 km] west), Ilesha, and Ondo highways and approximately 218 kms northeast of Lagos with a total land mass of 1,791 km^2^. Geographically, it lies within latitudes 7°28′ N and 7°46′ N and longitudes 4°36′ E and 4°56′ E. It falls under the Ife Federal Constituency and Osun East Senatorial District.

*Ilesa* is a town in Osun State in southwest Nigeria as well as the name of a historic kingdom centred in that city (also known as Ijesha). Its geographical coordinates are 7° 37′ 0″ N, 4° 44′ 0″ E. It lies in the Yoruba Hills at the intersection of roads from Ile-Ife, Osogbo, and Akure. It falls under the Ijesa South Federal Constituency and Osun East Senatorial District.

*Osogbo*: Also spelled Oshogbo, this town is the capital of Osun State in southwestern Nigeria. It has a population of 395,500 people and is bordered by Ikirun, Ilesa Ede, Egbedore, and Iragbiji. It is easily accessible from anywhere in the state, with coordinates of 7°46′ N 4°34′ E and a total landmass of 2875 km^2^. The city had a population of 156,694 people according to the 2006 census, which increased to 772,000 in 2023^11^. It falls under the Osogbo Federal Constituency and Osun Central Senatorial District.

### Target population

The study populations were traditional birth attendants (TBAs) who were registered and who had structures and birth centres within Osun State. A total of 426 TBA centres were estimated to be within these locations, i.e., the number of TBAs with centres.

### Inclusion criteria

The inclusion criteria for this study were TBAs within Gbogan, Ilesa, Osogbo and Ile-Ife who had physical structures within the area for delivery (because the study focused on the prevention and management of postpartum haemorrhage) and indicated willingness to participate in the study.

### Exclusion criteria

Auxiliary nurses, community health officers/extension workers, registered nurses/midwives or other skilled health workers who participated in deliveries were not considered TBAs. Additionally, the study excluded TBAs who had no delivery centres and who were unwilling to participate.

### Sample size determination

The sample size for the study was computed using Fisher’s formula as depicted below:

$${\text{n }} = \frac{{{\text{Z}}^{2} {\text{pq}}}}{{{\text{d}}^{2} }}$$

n = desired minimum sample size, Z = standard normal deviate (1.96) with 95% confidence interval, *P *= 20% (0.2) = Proportion of TBAs who have management protocol for PPH as reported by Bulndi et al.^21^

$${\text{q }} = { 1} - {\text{ p}} = { 1} - 0.{2 } = \, 0.{8}$$

$${\text{n}} = \left( {{1}.{96}^{{2}} \times \, 0.{2 } \times \, 0.{8}} \right)/0.0{5}^{{2}}$$

n = 245.8 = **246** taking into account 10% attrition or nonrespondent rates, i.e., 10% of 246 = 24.6 = 25.

Hence, the required sample size is 246 + 25 = **271**.

Therefore, the sample size for this study is 271 traditional birth attendants.

### Sampling technique

The study adopted a multistage sampling technique comprising stratified, purposive and proportionate sampling techniques as described by Adeyemi^22^ and modified by the authors. In the first stage, the state was captured based on the 3 existing strata (3 senatorial districts), namely, Osun Central, Osun West, and Osun East. Subsequently, one area was purposively selected from the Osun Central and Osun West senatorial districts, while two areas were selected from the Osun East senatorial district (the largest senatorial district in terms of landmass). The areas were selected due to the availability of TBAs. Thus, Osogbo represented Osun Central, Gbongan was selected to represent Osun West, while Ile-Ife and Ilesa were selected to represent Osun East. A proportionate sampling technique was adopted in the determination of the number of TBA centres to be selected from the sample frame of each of the four (4) selected study areas [Gbongan (39 TBAs), Ile-Ife (96 TBAs), Ilesa (139 TBAs) and Osogbo (152 TBAs)]. One respondent was interviewed from each designated centre.

A community mapping exercise was conducted to identify and enumerate all TBA centres across the four locations of Ilesa, Osogbo, Gbongan and Ile-Ife. Only those centres that met the criteria and were willing to participate in the study were recruited into the study. Proportionate allocation of Traditional Birth Attendants Centres in selected areas is shown below.

$$\begin{gathered} {\text{Gbongan }} = \frac{{39}}{{426}} \times { 271} = { 24}.{8}\sim {25} \hfill \\ \end{gathered}$$

$$\begin{gathered} {\text{Ile-}}{\text{Ife }} = \frac{{96}}{{426}} \times { 271} = { 61}.{1}\sim {61} \hfill \\ \end{gathered}$$

$$\begin{gathered} {\text{Ilesa }} = \frac{{139}}{{426}} \times { 271} = { 88}.{4}\sim {88} \hfill \\ \end{gathered}$$

$$\begin{gathered} {\text{Osogbo }} = \frac{{152}}{{426}} \times { 271} = { 96}.{7}\sim {97} \hfill \\ \end{gathered}$$

### Definition of variables

The following operational terms were adopted in the study and form the basis on which the instrument was developed:

- *Knowledge*: facts or information on postpartum haemorrhage acquired by traditional birth attendants through experience or education.
- *Good knowledge*: A comprehensive and deep understanding of postpartum haemorrhage. It includes the ability to apply information on postpartum haemorrhage in a meaningful way and make informed decisions.
- *Poor knowledge*: lacking information on or uninformed about postpartum haemorrhage.
- *Good Management*: maximizing one’s potential and best utilization of one’s unique skills for the identification, intervention and care of people with postpartum haemorrhage.
- *Poor management*: improper handling of postpartum haemorrhage leading to negative outcomes.
- *Practices*: actual application or use of an idea or belief that promotes or aids exclusive breastfeeding among nursing mothers.
- *Preventive practice*: practice intended to protect, promote or maintain the health and well-being of mothers and help to prevent disability or death arising from postpartum haemorrhage.
- *Postpartum haemorrhage*: severe vaginal bleeding after childbirth usually caused by loss of tone in the uterine muscles, a bleeding disorder or the placenta failing to come out completely or tearing.

### Instrument for quantitative data collection

A semistructured, self-administered questionnaire was used for data collection in this study. The questionnaires were adapted by the authors from other studies^21,23^. The components of the questionnaire were categorized into five (5) segments:

- *Section* A contained questions that assessed the sociodemographic information of the respondents (TBAs), including age at last birthday, gender, educational level, ethnicity, religion, year of practice, year of establishment, and mode of training.
- *Section* B contained questions to determine the participants’ knowledge of the causes and warning signs of PPH. Knowledge of PPH was scored based on ten (10) items. TBA knowledge was categorized into poor or good knowledge. Every correct answer, that is, a ‘yes’, was scored 1 point for a total of 10 points for knowledge of the causes and warning signs of postpartum haemorrhage. The 50th percentile (the mean) was used as the baseline for scoring poor/good knowledge.
- *Section* C contained questions to assess the management practices of TBAs towards PPH. Management based on the existing protocol was scored based on ten (10) items. The existing protocols were categorized into poor and good management practices. ‘Always’ and ‘sometimes’ responses were scored 1 point for a total of 10 points, while ‘rarely’ and ‘never’ responses were scored ‘0’ points for management practices of postpartum haemorrhage. The 50th percentile was used as the baseline for scoring poor/good management practices. A four (4) point Likert scale was adopted in this study to eliminate neutrality. Also, it is best use in situations in which a specific user opinion is essential^24^.
- *Section* D assessed the preventive practices of TBAs against PPH. Preventive practices were scored based on nine (9) items. Responses were categorized into inadequate and adequate preventive practices. Every correct answer, that is, a ‘yes’ response, was scored 1 point for a total of 9 points for adequate preventive practices for postpartum haemorrhage. The 50th percentile was used as the baseline for scoring inadequate/adequate preventive practices.
- *Section* E assessed the factors influencing the management practices of PPH among TBAs.

### Validity and reliability of the instrument

This study ensured external and content validity. The researchers and two other experts in the field of study closely examined the items in the questionnaire to ensure that they could accurately measure the intended variables. A pretest was conducted among TBAs in other locations outside the study settings (Ede and Ipetumodu). Relevant adjustments were made before the final administration of the questionnaire to the research population. The test–retest method was used to assess the reliability of the instrument. The internal consistency of the items showed an intraclass correlation coefficient of 0.8.

### Data collection procedure

The period for data collection was twelve (12) weeks from April to June 2022. Data were collected through self-administration of the questionnaires. The questionnaire was translated to Yoruba and was as simple and clear as possible with the targeted sections and questions. The researcher visited only TBA centres that conducted deliveries. Respondents were met at their respective centres and informed of the study. Each participant was equally informed about the purpose of the study, and guidelines for the completion of the questionnaire were explained to the participants, who were asked to tick where appropriate. The data collection lasted for 3 months.

### Data management and statistical analyses

After data collection, the questionnaires were properly checked for completeness. The collected data were entered manually and analysed using IBM Statistical Product and Service Solutions version 24 (SPSS, Inc. USA). Frequency tables, percentages, charts and descriptive statistics (mean and standard deviation) were used to report the results.

Inferential statistics chi-square (χ2) was used to test for associations between the independent variables (respondents’ sociodemographic variables) and outcome variables (management and preventive practices). Additionally, a multiple logistic regression model was used to assess predictors of the preventive and management practices of TBAs towards PPH. Statistical significance was one-tailed, with a p value less than 0.05 for all inferential analyses.

### Ethical considerations

The study was conducted in conformity with the Declaration of Helsinki, and the protocol was approved and ethical clearance was obtained from the Osun State Health Research Ethical Committee (**PROTOCOL NUMBER: OSHREC/PRS/569 T/261)**. Informed consent was obtained from individual participants before the commencement of data collection. In addition, respondents were informed of their right to voluntarily participate or withdraw from the study at any stage without adverse consequences. All methods were performed in accordance with the relevant guidelines and regulations.

## Results

A total of 271 questionnaires were administered, of which only 220 were correctly completed, returned and subsequently analysed, for a response rate of 81%.

The socio-demographics of the respondents are presented in Table 1. The ages of the respondents ranged from 20 to above 75 years, with a mean age of 53.3 ± 10.1. Most (164, 74.5%) of the TBAs were females. With regard to years of experience, the majority (154, 70%) had more than 20 years of practice, and few (7.3%) had less than 10 years of practice. The educational status of the respondents showed that those with secondary education were the most represented (103, 46.8%), while few reported no education (17, 7.7%). The majority of the respondents were predominantly Christian (140, 63.7%) of Yoruba ethnicity (198, 90%). Regarding the operational facts of the birth centres, 179 (81.4%) recorded fewer than 10 births per month, and approximately half (112, 50.9%) of the TBA centres were established in the twentieth century. Nearly two-thirds (145, 65.9%) of the respondents received formal training on pregnancy care and delivery, and all 145 (100.0%) reported receiving their training from a missionary organization. Most (157, 71.4%) of the TBAs were clerics, while the others were herbalists (63, 28.6%).

**Table 1:** Sociodemographic Characteristics of Traditional Birth Attendants.

Variables	Frequency (n) = 220	Percent (%)
Age		
Less than 40	19	8.6
40–59	111	50.5
60 and above	90	40.9
Mean ± SD	53.3 ± 10.1	
Range	(20,75)	
Gender		
Male	56	25.5
Female	164	74.5
Years of practice		
Less than 10	16	7.3
10–20	50	22.7
20 and above	154	70
Mean ± SD	22.3 ± 9.8	
Educational level		
Primary	71	32.3
Secondary	103	46.8
Tertiary	29	13.2
None	17	7.7
Ethnicity		
Yoruba	198	90
Igbo	5	2.3
Others	17	7.7
Religion		
Christianity	140	63.7
Islam	39	17.7
Traditional	41	18.6
Average no. of births per month		
Less than 10	179	81.4
10 and above	41	18.6
Mean ± SD	7 ± 3.8	
Year of establishment		
Before 1990	45	20.5
1990–1999	63	28.6
2000 and above	112	50.9
Received formal training on pregnancy care and delivery		
Yes	145	65.9
No	75	34.1
If yes, type		
Missionary	145	100.0
Year of training		
Before 1990	15	6.8
1990–1999	24	10.9
2000 and above	106	48.3
Type of TBA		
Cleric	157	71.4
Herbalist	63	28.6

**Figure 2:**
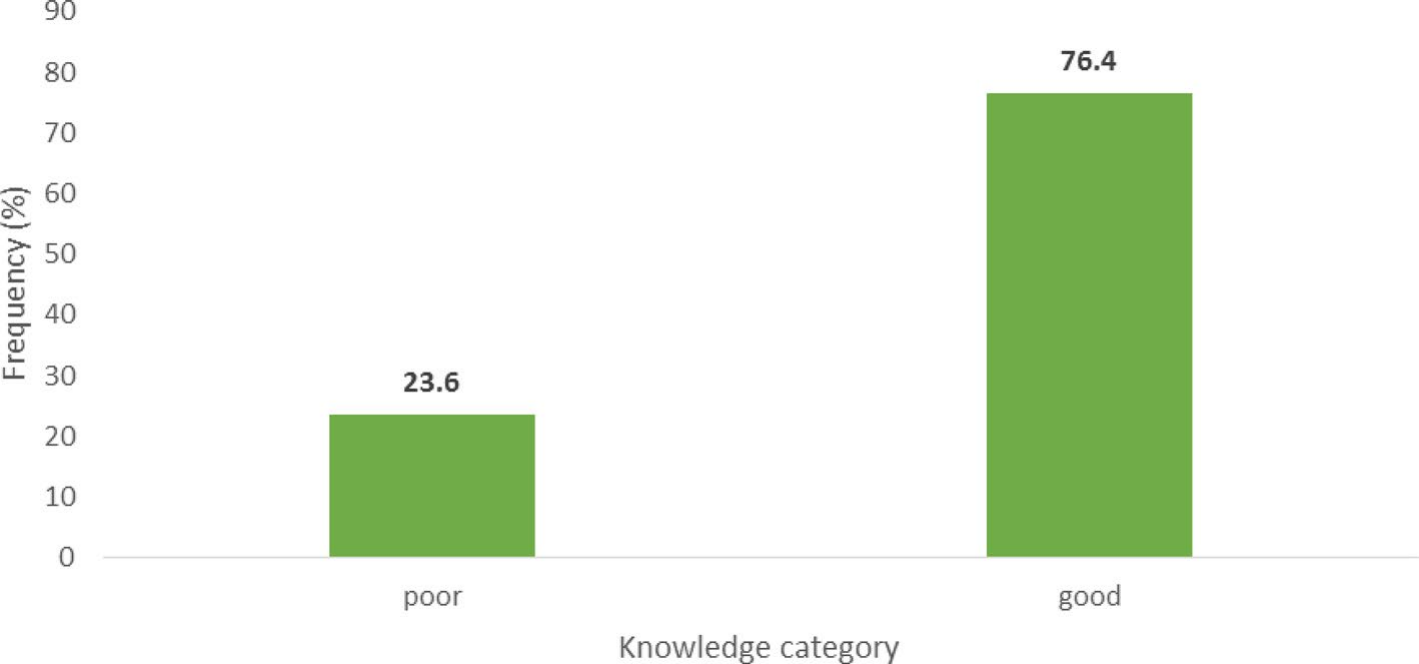
Summary Level of Respondents’ Knowledge of the Causes and Warning Signs of Post-Partum Hemorrhage.

Table 2a shows the respondents’ knowledge of the causes and warning signs of postpartum haemorrhage. Most of the respondents reported the following as causes and warning signs of PPH: bad obstetric history or previous stillbirth, 149 (67.7%); hypertension or fits, 191 (86.8%); anaemia or fatigue, 168 (76.4%); cessation of foetal movement, 168 (76.4%); abnormal foetal positioning, 123 (55.9%); sepsis or postpartum abdominal pain, 197 (89.5%); light bleeding or spotting, 177 (80.5%); haemorrhage, 176 (80.0%); and obstructed or prolonged labour, 197 (89.5%). In contrast, multiple pregnancy or large abdomen was largely unknown to the TBAs (169, 76.8%) to cause and forewarn of postpartum haemorrhage. In summary, an average of 72% of the TBAs had knowledge of postpartum haemorrhage. As shown in Fig. 2, most (168, 76.4%) of the TBAs had good knowledge of the causes and warning signs of postpartum haemorrhage, while 52 (23.6%) of them had poor knowledge.

**Table 2:** Respondents’ Knowledge and Preventive Practices of TBAs for Postpartum Haemorrhage.

Items	Frequency (n)	Percent (%)
*a. Respondents’ Knowledge of the Causes and Warning Signs of Postpartum Haemorrhage*		
Previous bad obstetric history or abdominal scars or previous stillbirth		
Yes	149	67.7
No	28	12.7
I don’t know	43	19.6
Hypertension, headache, swelling or fits		
Yes	191	86.8
No	5	2.3
I don’t know	24	10.9
Anaemia, pallor, fatigue or breathlessness		
Yes	168	76.4
No	40	18.2
I don’t know	12	5.4
Cessation of foetal movement or baby does not move		
Yes	168	76.4
No	34	15.5
I don’t know	18	8.1
Abnormal lie or position of foetus		
Yes	123	55.9
No	78	35.5
I don’t know	19	8.6
Sepsis or foul-smelling discharge or postpartum abdominal pain		
Yes	197	89.5
No	11	5
I don’t know	12	5.5
Light bleeding or spotting		
Yes	177	80.5
No	32	14.5
I don’t know	11	5
Haemorrhage or heavy bleeding		
Yes	176	80
No	34	15.5
I don’t know	10	4.5
Multiple pregnancy or large abdomen		
Yes	38	17.3
No	169	76.8
I don’t know	13	5.9
Obstructed or prolonged labour		
Yes	197	89.5
No	15	6.8
I don’t know	8	3.6
*b. Preventive Practices of TBAs for Postpartum Haemorrhage*		
Practice		
Administer I.M. Oxytocin 10 i.u. immediately after the delivery of the baby		
Yes	66	30
No	154	70
Administer I.M. Ergometrine 0.2 mg (2 cc) after the delivery of the baby		
Yes	42	19.1
No	178	80.9
Administer Misoprostol 600 mcg by mouth before the delivery of the placenta		
Yes	39	17.7
No	181	82.3
Do uterine massage after delivery of the Placenta		
Yes	97	44.1
No	123	55.9
Delay clamping/cutting the cord after delivery of the baby		
Yes	183	83.2
No	37	16.8
Stimulate the nipples of the woman to prevent		
PPH		
Yes	171	77.7
No	49	22.3
Encourage the woman to empty her bladder Regularly		
Yes	179	81.4
No	41	18.6
Deliver the placenta by cord-controlled traction		
Yes	65	29.5
No	155	70.5
Allow the woman to deliver the placenta herself		
Yes	180	81.8
No	40	18.2

**Figure 3:**
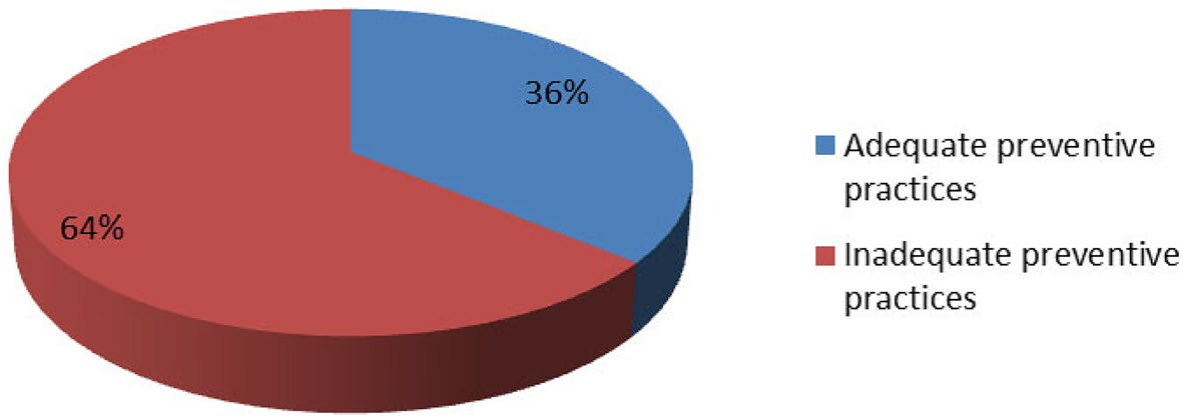
Summary of Preventive Practices of TBAs toward Post-Partum Hemorrhage.

Table 2b shows the preventive practices of TBAs towards postpartum haemorrhage of mothers, which included taking recommended medications and physical practices before and after delivery of the baby. Regarding medications, the TBAs largely reported not administering the following medications: I. M Ergometrine 0.2 mg (2 cc) immediately after delivery, 178 (80.9%); I.M. M oxytocin 10 i.u., 154 (70%); and misoprostol 600 mcg by mouth before delivery, 181 (82.3%). Conversely, the results showed mixed outcomes on the performance of physical practices. Specifically, 183 (83.2%) indicated delayed clamping or cutting of the baby’s cord after delivery, 171 (77.7%) indicated stimulating the woman’s nipples to prevent PPH, 65 (29.5%) delivered the placenta by cord-controlled traction, 39 (17.7%) performed uterine massage after delivery of the placenta, and 179 (81.4%) encouraged mothers to regularly empty their bladders. On average, approximately 51.6% of the TBAs had at least one preventive practice for postpartum haemorrhage. Figure 3 shows that a high proportion (141, 64.1%) of the respondents had inadequate preventive practices, while 79 (35.9%) had adequate preventive practices.

**Figure 4:**
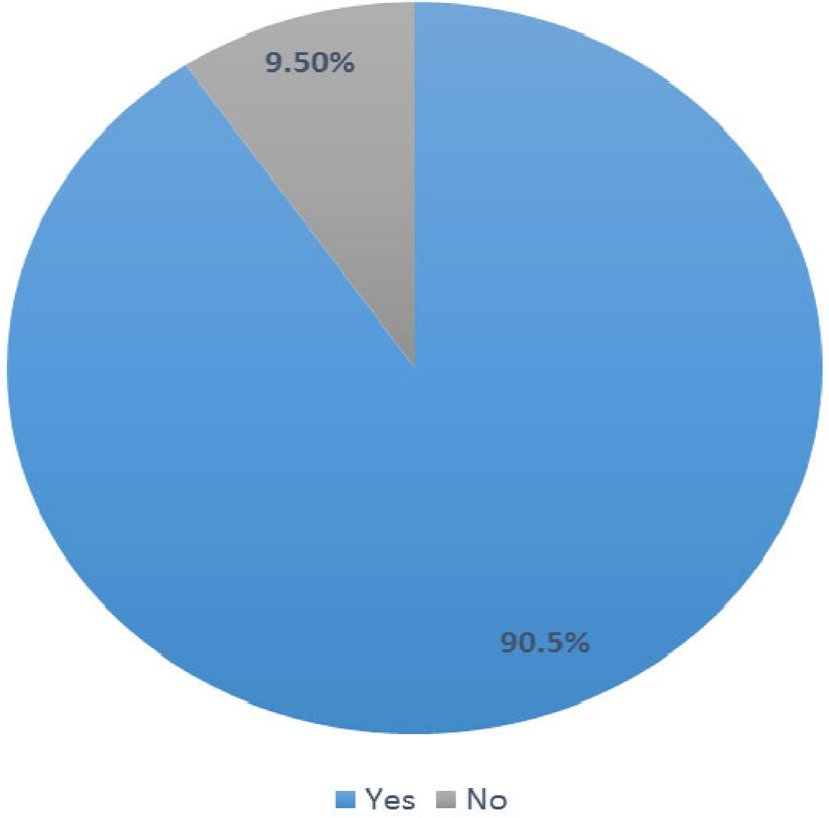
Ever Managed Post-Partum Hemorrhage.

**Figure 5:**
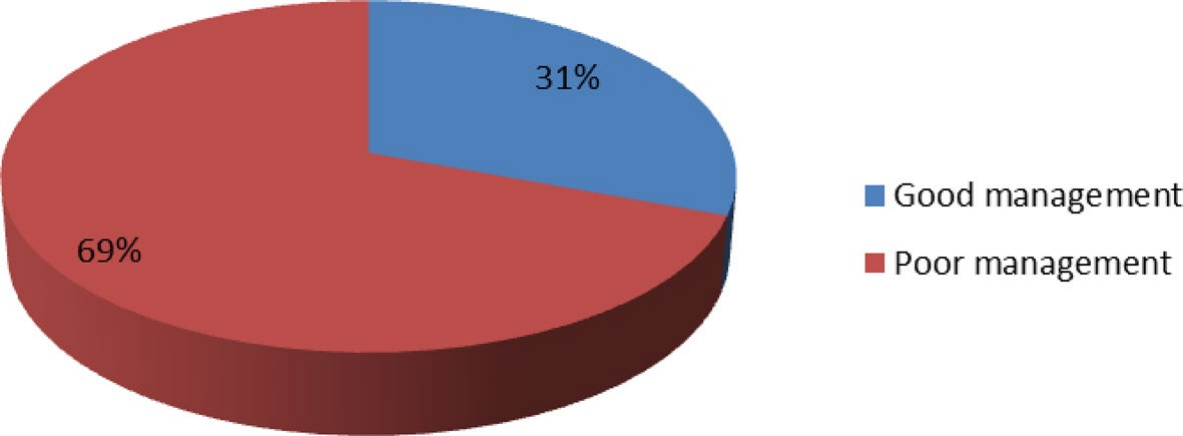
Summary of Management Practices of TBAs toward Post-Partum Haemorrahage.

**Figure 6:**
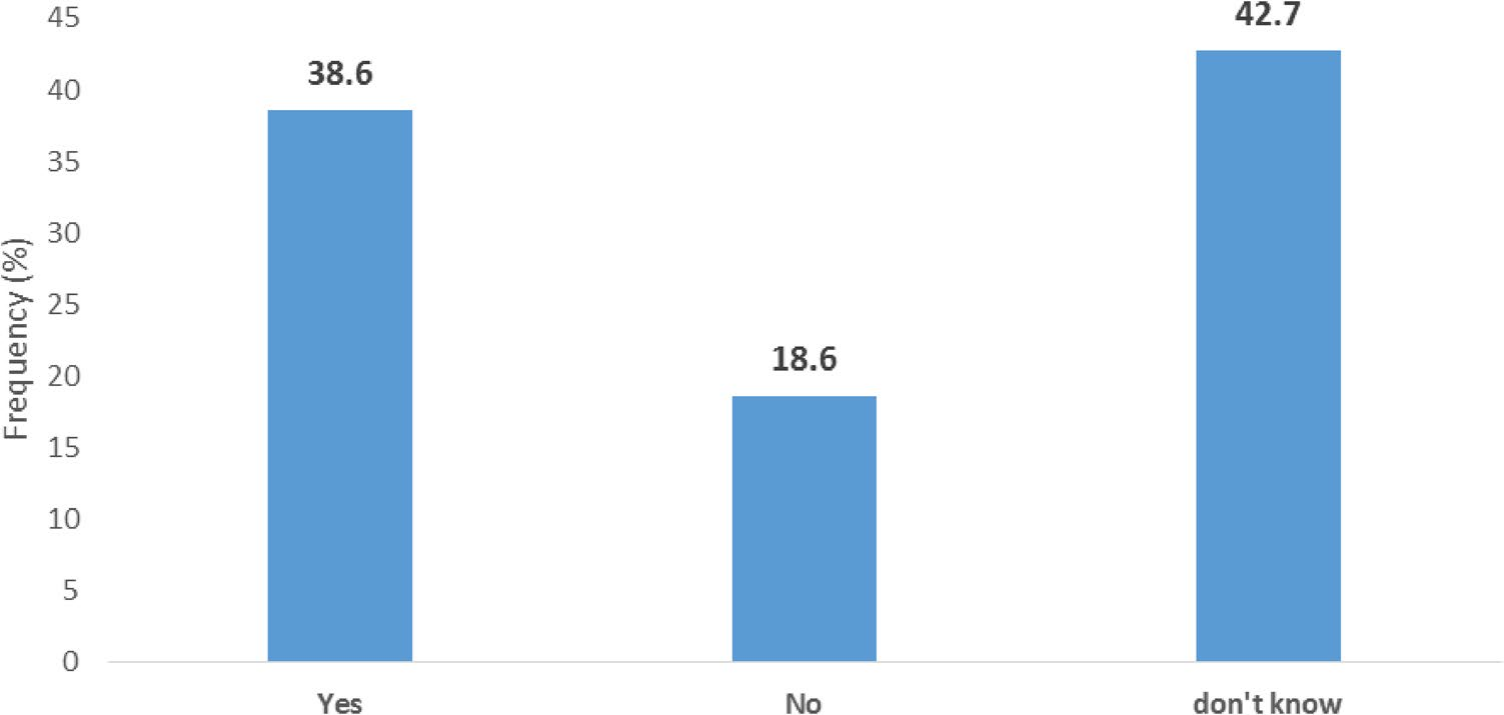
TBAs’ Knowledge of Existing protocols for the Management of PPH.

As depicted in Fig. 4, a huge majority (199, 90.5%) of the TBAs had previously managed postpartum haemorrhage, while 21 (9.5%) had not managed it previously. Table 3 shows the management practices adopted by the TBA centres towards women with postpartum haemorrhage. TBA centres reported always practising the following: the use of appropriate sterile instruments, 195 (98%); the use of herbs or flowers for controlling bleeding, 65 (32.7%); uterine massage, 65 (32.7%); giving mothers concoctions, 62 (31.2%); use of anointing and other forms of oils, 57 (28.6%); and manual extraction, 31 (15.6%). One hundred and twenty-four (62.3%) of the TBA centres sometimes utilized herbs to stimulate contractions, while 23 (11.6%) sometimes made use of cooking sticks or chains of beads down the mother’s throat. However, the following were never practised by a high proportion of the TBAs centres: the use of appropriate instruments that were not sterile, 194 (97.5%); the use of misoprostol, 171 (85.9%); doing nothing, 168 (84.4%); the use of intravenous fluids, 142 (71.4%); the use of oxytocin, 137 (68.8%); and referral to a health facility, 110 (55.3%). Most of the existing protocols frequently used by the TBA centres were mental status monitoring (193, 97%), vital signs and oxygen saturation (150, 75.4%), asking for help (143, 71.9%), and urinary catheter (106, 53.3%). Existing protocols that were rarely used were blood transfusion (185, 93.0%), fluid augmentation (160, 80.4%), prevention of hypothermia (134, 67.3%), medication administration (132, 66.3%), bimanual uterine compression (127, 63.8%), and strong uterine massage (107, 53.8%). Figure 5 shows that of the 199 respondents who had previously managed postpartum haemorrhage, a high proportion (138, 69.3%) of the respondents had poor management practices, while 61 (30.7%) had good management practices with regard to PPH. As displayed in Fig. 6, a high proportion (94, 42.7%) of the TBAs did not know whether there were existing protocols for the management of PPH. Eighty-five (38.6%) knew of existing protocols, while 41 (18.6%) said that no protocols for PPH management existed. The most cited existing protocols were manuals from schools and conferences, followed by the use of incantation and herbs.

**Table 3:** Management Practices of TBAs for Postpartum Haemorrhage (n = 199).

Practices	Always	Sometimes	Rarely	Never
Use of appropriate sterile instrument	195(98)	0(0)	0(0)	4(2)
Appropriate instrument but not sterile	3(1.5)	1(0.5)	1(0.5)	194(97.5)
Use of herbs for stimulation of contractions	37(18.6)	124(62.3)	2(1.0)	36(18.1)
Use of herbs or flowers for control of bleeding	65(32.7)	2(1)	4(2)	128(64.3)
Cooking stick or chain of beads down mother’s throat	3(1.5)	23(11.6)	2(1.0)	171(85.9)
Giving mothers concoctions	62(31.2)	3(1.5)	4(2)	130(65.3)
Use of anointing and other forms of oil	57(28.6)	5(2.5)	4(2)	133(66.8)
Uterine massage	65(32.7)	8(4)	3(1.5)	123(61.8)
Manual extraction	31(15.6)	3(1.5)	17(8.5)	148(74.4)
Use of oxytocin	5(2.5)	28(14.1)	29(14.6)	137(68.8)
Use of misoprostol	4(2)	19(9.5)	5(2.5)	171(85.9)
Refer to health facility	4(2)	52(26.1)	33(16.6)	110(55.3)
Use of intravenous fluids	5(2.5)	26(13.1)	26(13.1)	142(71.4)
Do nothing	8(4)	15(7.5)	8(4)	168(84.4)
Management using existing protocols				
Asking for help (communication)	143(71.9)	39(19.6)	16(8.0)	1(0.5)
Mental status monitoring	193(97.0)	3(1.5)	0(0)	3(1.5)
Vital signs and oxygen saturation	150(75.4)	10(5)	1(0.5)	38(19.1)
Urinary catheter	106(53.3)	29(14.6)	26(13.1)	38(19.1)
Fluid augmentation	11(5.5)	3(1.5)	25(12.6)	160(80.4)
Blood transfusion	11(5.5)	2(1.0)	1(0.5)	185(93.0)
Prevention of hypothermia	55(27.6)	5(2.5)	5(2.5)	134(67.3)
Bimanual uterine compression	27(13.6)	37(18.6)	8(4)	127(63.8)
Strong uterine massage	28(14.1)	28(14.1)	36(18.1)	107(53.8)
Medication administration	34(17.1)	6(3.0)	27(13.6)	132(66.3)

### Association between TBAs’ sociodemographic characteristics and management practices

The results revealed that the TBA type (*P* = 0.031), gender (*P* = 0.029), educational level (*P* = 0.035) and average number of births per month (*P* = 0.001) showed significant associations with TBAs’ management practices (Table 4).

**Table 4:** Association between TBAs’ Sociodemographic Characteristics and Management Practices (n = 199).

	Poor	Good	Total	${{\varvec{\chi}}}^{2}$	*P* value
TBA type					
Cleric	102(74.5)	35(25.5)	137	4.649^y^	0.031*
Herbal	36(58.1)	26(41.9)	62		
Age					
Less than 40	7(50)	7(50)	14	5.224	0.07
40–59	65(65.7)	34(34.3)	99		
60 and above	66(76.7)	20(23.3)	86		
Gender					
Male	28(56)	22(44)	50	4.789^y^	0.029*
Female	110(73.8)	39(26.2)	149		
Years of practice					
Less than 10	8(72.7)	3(27.3)	11	2.392	0.302
10–19	27(60)	18(40)	45		
20 and above	103(72)	40(28)	143		
Educational level					
Primary	49(72.1)	19(27.9)	68	8.595	0.035*
Secondary	67(72)	26(28)	93		
Tertiary	10(43.5)	13(56.5)	23		
None	12(80)	3(20)	15		
Religion					
Christianity	82(65.6)	43(34.4)	125	2.613	0.271
Islam	26(72.2)	10(27.8)	36		
Traditional	30(78.9)	8(21.1)	38		
Average no. of births per month					
Less than 10	102(63.8)	58(36.2)	160	10.724	0.001*
10 and above	36(92.3)	3(7.7)	39		
Year of establishment					
Before 1990	32(74.4)	11(25.6)	43	0.873	0.646
1990–1999	40(70.2)	17(29.8)	57		
2000 and above	66(66.7)	33(33.3)	99		
Received formal training on pregnancy care and delivery					
Yes	96(72.7)	36(27.3)	132	1.662^y^	0.197
No	42(62.7)	25(37.3)	67		
Year of training					
Before 1990	11(78.6)	3(21.4)	14	1.336	0.513
1990–1999	14(82.4)	3(17.6)	17		
2000 and above	71(70.3)	30(29.7)	101		

${\chi }^{2}:$ Chi-square test; ^y^: Yates correction; *:*P* < 0.005.

### Association between TBAs’ sociodemographic characteristics and preventive practices

The results revealed that the TBA type (*P* < 0.001), average number of births per month (*P* = 0.003) and receipt of formal training (*P* = 0.005) showed a significant association with TBAs’ preventive practices (Table 5).

**Table 5:** Association between TBAs’ Sociodemographic Characteristics and Preventive Practices.

	Preventive Practices (n = 220)				
	Inadequate	Adequate	Total	${\chi }^{2}$	*P* value
TBA type					
Cleric	114(72.6)	43(27.4)	157	16.026^y^	< 0.001*
Herbal	27(42.9)	36(57.1)	63		
Age					
Less than 40	11(57.9)	8(42.1)	19	0.475	0.789
40–59	73(65.8)	38(34.2)	111		
60 and above	57(63.3)	33(36.7)	90		
Gender					
Male	32(57.1)	24(42.9)	56	1.197^y^	0.274
Female	109(66.5)	55(33.5)	164		
Years of practice					
Less than 10	11(68.8)	5(31.2)	11	0.248^y^	0.883
10–19	31(62)	19(38)	45		
20 and above	99(64.3)	55(35.7)	143		
Educational level					
Primary	41(57.7)	30(42.3)	71	7.585	0.055
Secondary	73(70.9)	30(29.1)	103		
Tertiary	14(48.3)	15(51.7)	29		
None	13(76.5)	4(23.5)	17		
Religion					
Christianity	86(61.4)	54(38.6)	140	4.346	0.114
Islam	23(59)	16(41)	39		
Traditional	32(78)	9(22)	41		
Average no. of births per month					
Less than 10	106(59.2)	73(40.8)	179	8.807y	0.003*
10 and above	35(85.4)	6(14.6)	41		
Year of establishment					
Before 1990	27(60.0)	18(40.0)	45	1.407	0.495
1990–1999	38(60.3)	25(39.7)	63		
2000 and above	76(67.9)	36(32.1)	112		
Received formal training on pregnancy care and delivery					
Yes	103(71.0)	42(29.0)	145	8.047y	0.005*
No	38(50.7)	37(49.3)	75		
Year of training					
Before 1990	11(73.3)	4(26.7)	15	0.284	0.868
1990–1999	16(66.7)	8(33.3)	24		
2000 and above	76(71.7)	30(28.3)	106		

${\chi }^{2}:$ Chi-square test; ^y^: Yates correction; *:*P* < 0.005.

### Logistic regression model

As shown in Table 6, the logistic regression model was used to determine the factors that influenced the preventive and management practices of the TBAs. The significant factors in the bivariate analysis were incorporated into the regression model.

**Table 6:** Predictors of Preventive and Management Practices of TBAs towards PPH.

	B	*p* value	OR	95% C.I	
				Lower	Upper
Preventive practices (n = 220)					
TBA type (Cleric)	1.443	0.003*	**4.23**	1.64	10.90
Average no. of births monthly (≥ 10)	− 1.78	< 0.001*	0.17	0.06	0.45
Received formal training (No)	0.114	0.805	1.12	0.45	2.78
Management practices (n = 199)					
TBA type (Cleric)	1.486	< 0.001*	**4.42**	2.03	9.61
Gender (female)	− 0.733	0.059	0.48	0.23	1.03
Tertiary ^ref^					
Secondary	− 1.456	0.006*	0.23	0.08	0.65
Primary	− 1.602	0.006*	0.20	0.07	0.63
None	− 2.244	0.009*	0.11	0.02	0.57
Average no. of births monthly (≥ 10)	− 2.111	0.001*	0.121	0.034	0.436

B, coefficient of regression; OR, odds ratio; 95% CI, 95% confidence interval.

*: *p* value < 0.05.

Significant values are in [bold].

TBAs’ preventive practices towards PPH were influenced by the TBA type (OR: 4.23; 95% CI: 1.64–10.90); clerical TBAs were 4 times more likely to have inadequate preventive practices than herbalist TBAs. The average number of monthly births (fewer than 10 births) was a protective predictor (OR: 0.17; 95% CI 0.60–0.45); the more (fewer than 10) monthly average births there were, the less likely (83%) an individual was to have inadequate preventive practices compared to having 10 or more monthly average births. Similarly, the TBAs’ management practices were also influenced by the TBA type (OR: 4.42; 95% CI: 2.03–9.61); the cleric TBAs were 4 times more likely to have good management practices than the herbalist TBAs. TBAs’ educational level and average number of monthly births were less likely predictors.

## Discussion

Postpartum haemorrhage remains a major cause of maternal mortality^1^. However, the reduction of maternal mortality is part of the Sustainable Development Goals, Goal 3 target, which is to reduce global maternal mortality to less than 70 per 100,000 live births per year by 2030^25^.

In the present study, the majority of the TBAs were in the age group of 40–59 years, with a mean age of 53.3 ± 10.1 years, and most of them were female. Approximately half the respondents had secondary education, with the majority having more than 20 years of experience’. They were largely of the Yoruba tribe, while the TBA type was largely clerics and a few herbalists. These findings were in consonance with a similar study conducted in other parts of Nigeria^21^.

Knowledge refers to the awareness or understanding of a subject matter (person or thing), such as objects (acquaintance knowledge), facts (propositional knowledge) or skills (procedural knowledge)^26^. The present study revealed that most (76.4%) of the TBAs had good knowledge of the causes and warning signs of postpartum haemorrhage, while a few (23.6%) of them had poor knowledge. This is similar to a study among nurse-midwives in Bayelsa State, Nigeria, which equally showed a high level of knowledge about PPH among nurse-midwives^11,27^ and similar knowledge among nurse-midwives of the prevention and management of PPH in Khartoum, Sudan^12,28^. The high level of knowledge of the respondents may be attributed to their formal education and previous training. However, in contrast to our study, a study in the Gambia revealed a huge gap in the knowledge of TBAs regarding the prevention, recognition and management of postpartum haemorrhage; TBAs did not know the causes of excessive blood loss^13,29^. In addition, a study in Tanzania revealed a low level of knowledge of danger signs for complications of pregnancy, such as PPH^15,30^. Furthermore, in an interventional study in Ogbomoso Oyo State Nigeria, the preintervention knowledge level among TBAs for the identification of PPH was low (7.2%)^16,31^.

Active management of the third stage of labour (AMSTL) is a common method adopted by skilled providers to reduce the incidence of PPH in clinical practice. If used at every birth, AMSTL would reduce PPH by 30–50%^32^. AMSTL involves the administration of a prophylactic, clamping and cutting of the umbilical cord and controlled-cord traction as interventions during the baby’s birth^32,33^. The present study revealed that a high proportion (69.3%) of the respondents who had previously managed PPH had poor management practices, while 30.7% had good management practices. The common management skills for the prevention and control of PPH employed by the respondents included uterine massage, the use of appropriate sterile instruments, urinary catheters, the use of herbs or flowers for the control of bleeding, the use of herbs for the stimulation of contractions, and giving mothers concoctions. However, the following were rarely practised by the respondents: the use of oxytocin, the use of misoprostol, fluid augmentation and blood transfusion. This report negated the findings of Babatunde et al.^21^ in which TBAs reported the early use of cord clamping after 3 min, emptying of the bladder during the third stage of labour, placing women in the Trendeleburg position and the use of uterotonics. The WHO revealed that one way that unskilled birth attendants (TBAs) could help to reduce PPH is through the use of oral intervention with misoprostol and oxytocin, which were included in the WHO’s Essential Medicines List. The WHO now supports the administration of misoprostol by nonskilled birth attendants at home deliveries^34^.

Similarly, in the bivariate analysis, four factors were associated with good management practices, including the TBA type, gender, education level and the average number of births per month, which all showed statistically significant associations with TBA management practices (*P* < 0.05). However, in the binary logistic regression, the TBA type was the only statistically significant predictor of good management practice towards PPH. A qualitative study by Ononge et al. in rural Uganda revealed poor management of PPH by TBAs, with some believing that vaginal bleeding after childbirth was a normal and cleansing process and practising the use of herbs. However, it was reported that some referred to the health facility or invited health workers to assist them^17,35^. Similarly, a study by Konje et al. in Tanzania reported the management of PPH by TBAs using various methods, including the use of herbal medicines and abdominal massage, with the majority referring their clients to a nearby health facility^15,36^. These studies were similar to the present study because they both conducted research in developing countries (resource-poor settings) that retain several traditional beliefs, including the preferred use of TBAs, and many misconceptions.

This study reveals that a high proportion (64.1%) of the respondents had inadequate preventive practices, while few (35.9%) had adequate preventive practices. In the bivariate analysis, three factors were associated with preventive practises, including the TBA type, average number of births per month and receipt of formal training, all of which showed statistically significant associations with TBAs’ preventive practices (*P* < 0.05). However, in the binary logistic regression, the TBA type and average number of births per month were the only statistically significant predictors of good preventive practices towards PPH. In contrast to our study, a cross-sectional study by Faiza et al. among nurse-midwives in Khartoum State, Sudan, revealed a mean score of 69% for preventive practice^18,37^. This may have occurred because the study was among nurse-midwives who were more educated, skilled and professionally trained than the TBAs in this study. In another study, Kaingu et al. emphasized some preventive practices, such as the use of herbal medicine among TBAs, in preventing and treating haemorrhage^19,38^.

### Policy implementation and the way forward

A healthy nation is a prosperous nation. As it is sometimes claimed that "health is money”, the health sector in Nigeria is one of the institutions that deserves strong governance and development. The Nigerian health industry is divided into two distinct health systems: the traditional therapeutic system of treatment and the modernized system in the West. Although customs are not recognized by the government, studies have revealed that they enjoy widespread support in society^22^. Research has also shown that TBAs were in existence prior to the development of modern medicine, and the current study showed that TBAs have long faced PPH issues, necessitating the development of their own traditional or local therapy methods for managing this complication^22^.

To reduce maternal mortality in Nigeria, it is imperative to increase women's access to trained delivery attendants. Training TBAs to manage PPH, the main cause of maternal mortality, especially with the use of oral misoprostol to supplement their local treatment techniques as in other developing countries, may help to reduce maternal death in developing nations such as Nigeria, where very few births are attended by SBAs and traditional health care is valued. This will go a long way towards achieving both quantitative and quality health care in Nigeria and in Osun State specifically and hence excellent governance and development in the health sector.

## Conclusion

In this study, we found that most TBAs are knowledgeable about the cause and warning signs of PPH in pregnant women. However, there are inadequate preventive practices and poor management practices despite a high level of knowledge. We also found that the predictors of management and preventive practices for PPH were the type of TBA and the type of training received, while educational level and the average number of monthly births were less likely predictors. Thus, it is recommended that supportive supervision, constant monitoring and evaluation with continuous health education and training can maximize the gains of TBAs. Targeted intervention using identified factors will also increase the effectiveness of TBAs.

## Data Availability

All data generated or analysed during this study are included in this published article [and its supplementary information files].

## Abbreviations

TBAs: Traditional birth attendants
PPH: Postpartum haemorrhage
